# Supplementary material for: Longitudinal Associations of Adherence to the Dietary World Cancer Research Fund/American Institute for Cancer Research (WCRF/AICR) and Dutch Healthy Diet (DHD) Recommendations with Plasma Kynurenines in Colorectal Cancer Survivors after Treatment
Source: Nutrients. 2022 Dec 3;14(23):5151. doi: 10.3390/nu14235151 (PMC9738623; doi:10.3390/nu14235151)
Supplement: Supplementary file 1 [file nutrients-14-05151-s001.zip › Supplementary Figure S1 NEW.pdf]

Kynurenines and ratios with neuroprotective properties

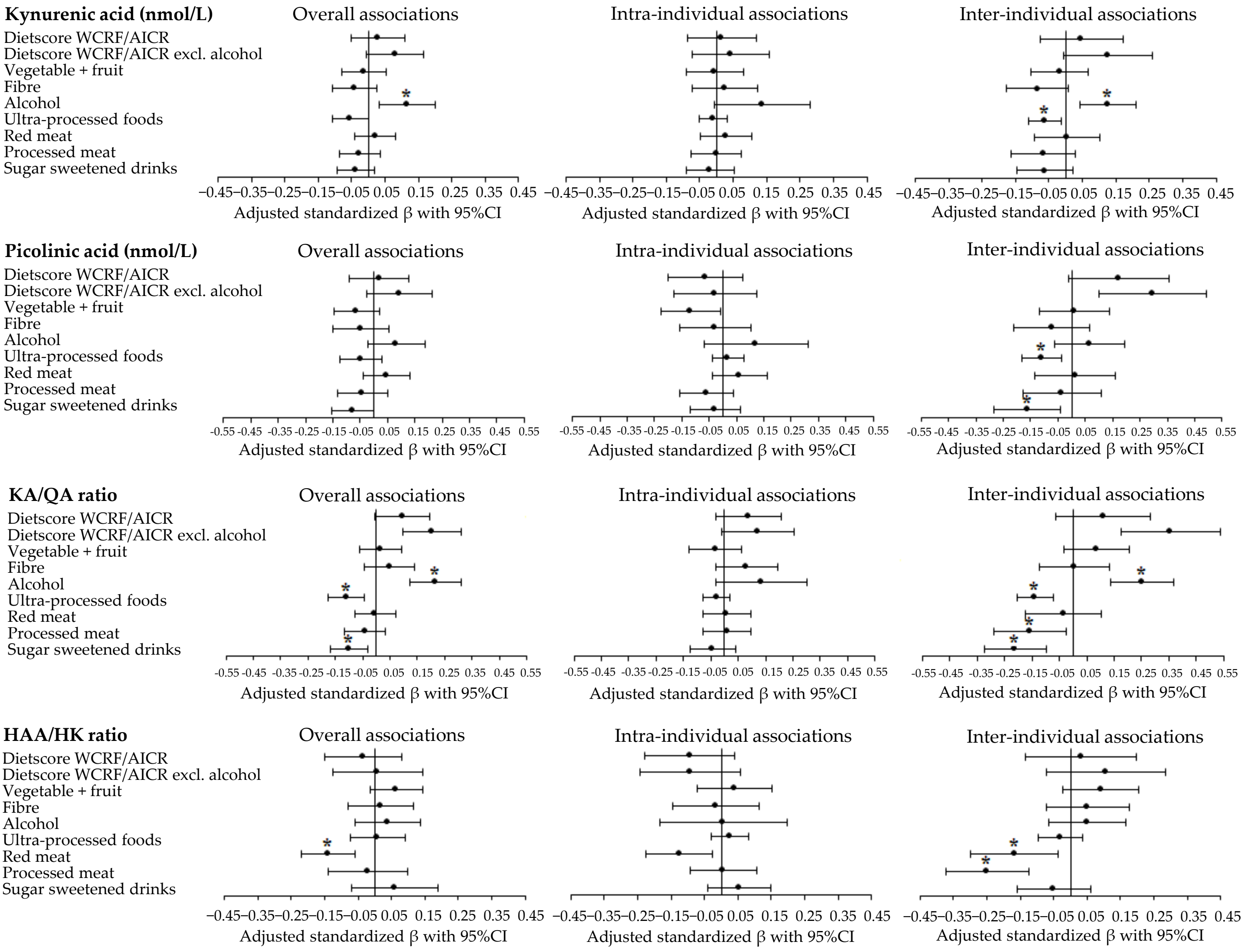

Kynurenines with less well characterized properties

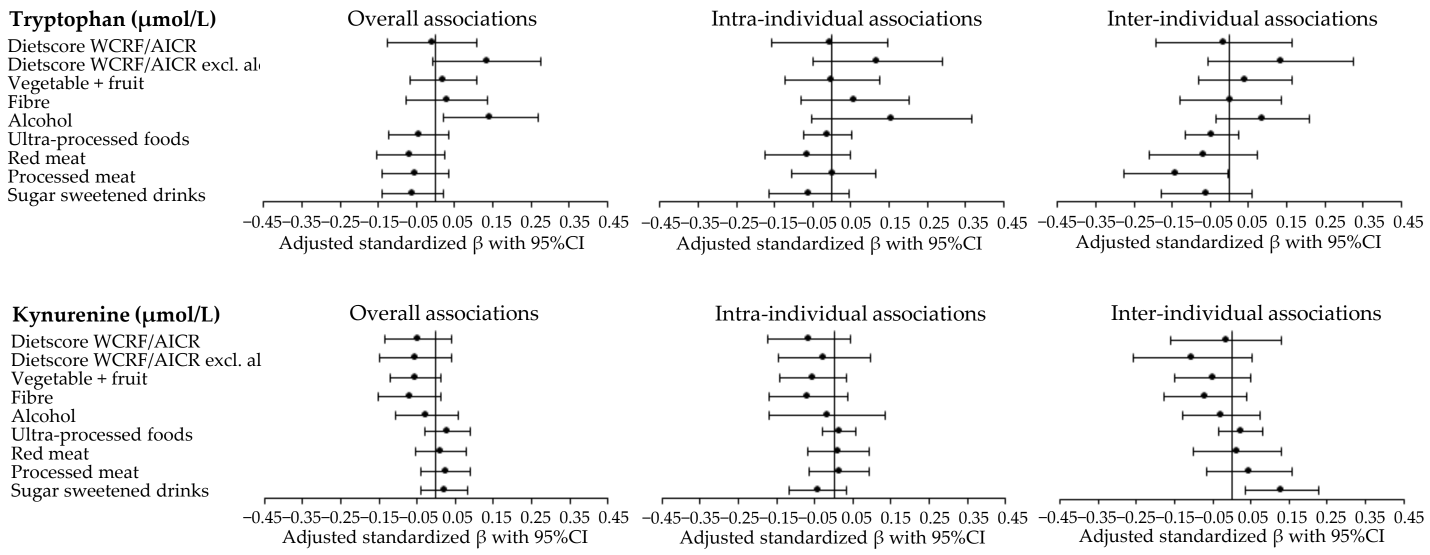

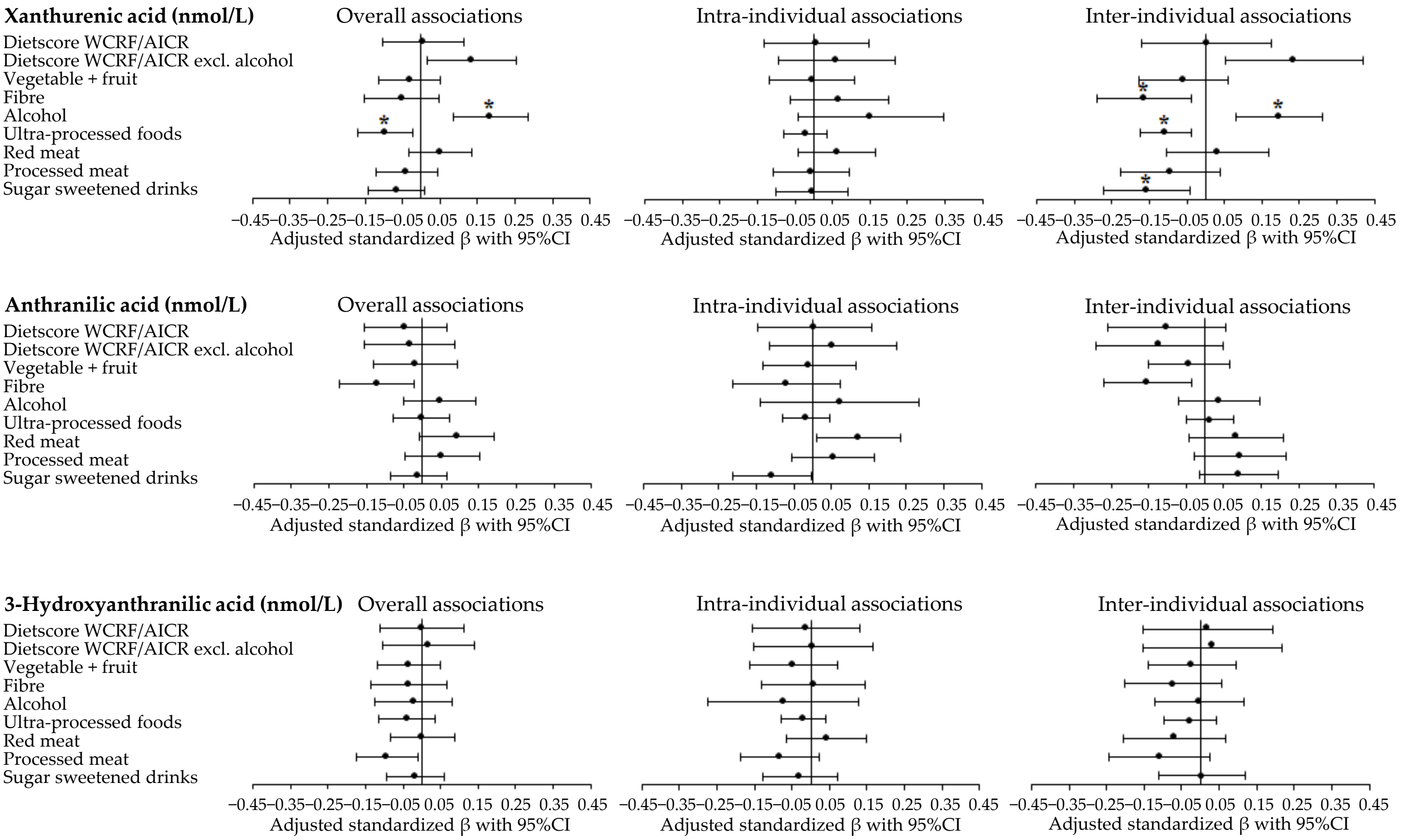

Kynurenines and ratios with neurotoxic properties

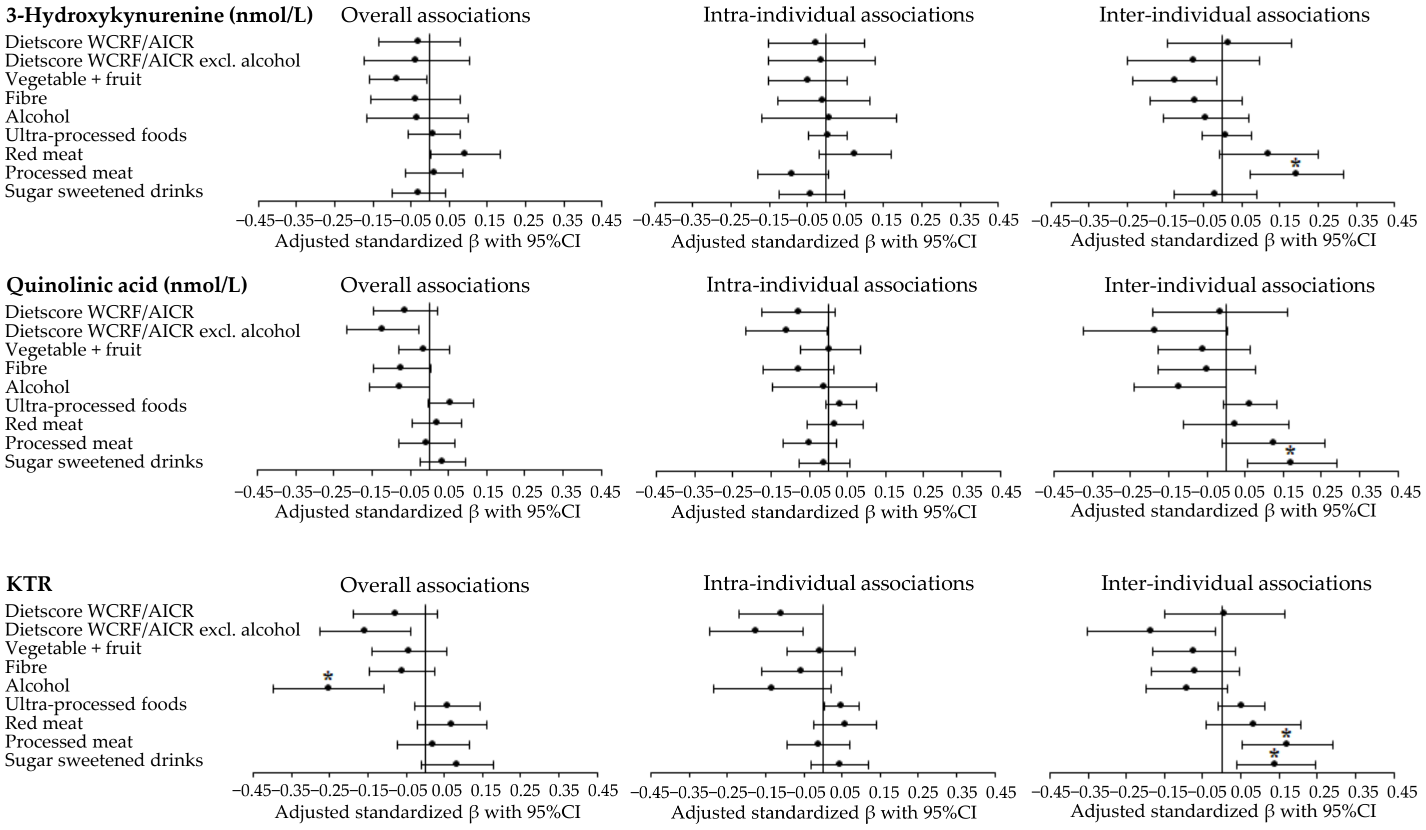

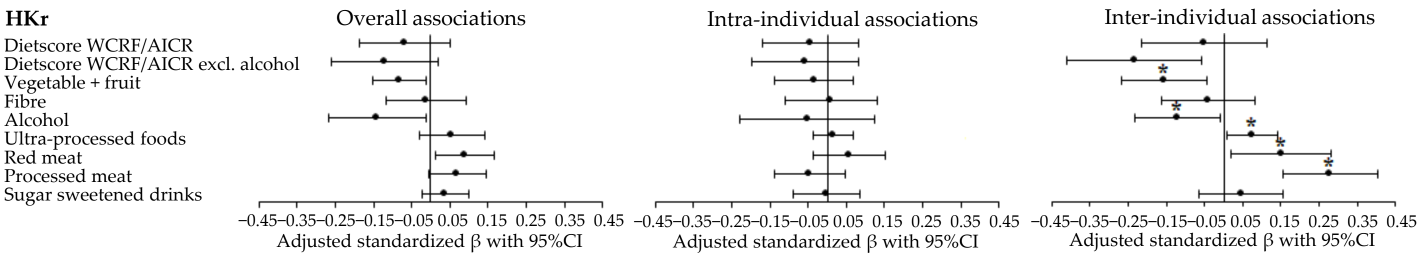

Supplementary figure S1. Forest plots of confounder-adjusted linear mixed models between adherence to the dietary WCRF/AICR recommendations and metabolites and ratios of the kynurenine pathway in colorectal cancer survivors. Analyses were adjusted for age at enrolment (years), sex (male, female), renal function (μmol/L), time since end treatment (weeks), chemotherapy (yes, no), comorbidities (0, 1, ≥2), stoma (yes, no), educational level (high, medium, low), BMI (kg/m²), total PA (hours/week), smoking status (current, former, never) and energy intake (kcal/week). A random slope was added to the model for dietscore WCRF/AICR with HK, HAA/HK, KTR, and HKr; for dietscore WCRF/AICR excl. alcohol with Trp, HK, HAA/HK, KTR, and HKr; for vegetable and fruit with KA, AA, KTR, and HKr; for dietary fibre with HK, KA, QA, and HKr; for alcohol with Trp, HK, KA, QA, KTR, and HKr; for ultra-processed foods with QA, HAA/HK, KTR, and HKr; for red meat intake with HK, AA, and KTR; and for processed meat intake with AA, Pic, QA, HAA/HK, and KTR; and for sugar-sweetened drink intake with HAA/HK, KTR, and HKr. The standardized β-coefficient can be interpreted as the amount of SD difference in kynurenine concentration/ratio according to 1SD difference in dietary score/intake.

* Statistically significant after FDR adjustment for multiple testing.

Abbreviations: KA, kynurenic acid; Pic, picolinic acid; KA/QA ratio, kynurenic-acid-to-quinolinic-acid ratio; HAA/HK ratio, hydroxyanthranilic-to-hydroxykynurenine ratio; Trp, tryptophan; Kyn, kynurenine; XA, xanthurenic acid; AA, anthranilic acid, HAA, 3-hydroxyanthranilic acid; HK, 3-hydroxykynurenine; QA, quinolinic acid, KTR, kynurenine-to-tryptophan ratio; HKr, hydroxykynurenine ratio.
