# Supplementary material for: Longitudinal Associations of Adherence to the Dietary World Cancer Research Fund/American Institute for Cancer Research (WCRF/AICR) and Dutch Healthy Diet (DHD) Recommendations with Plasma Kynurenines in Colorectal Cancer Survivors after Treatment
Source: Nutrients. 2022 Dec 3;14(23):5151. doi: 10.3390/nu14235151 (PMC9738623; doi:10.3390/nu14235151)
Supplement: Supplementary file 1 [file nutrients-14-05151-s001.zip › Supplementary Figure S2 NEW.pdf]

Kynurenines and ratios with neuroprotective properties

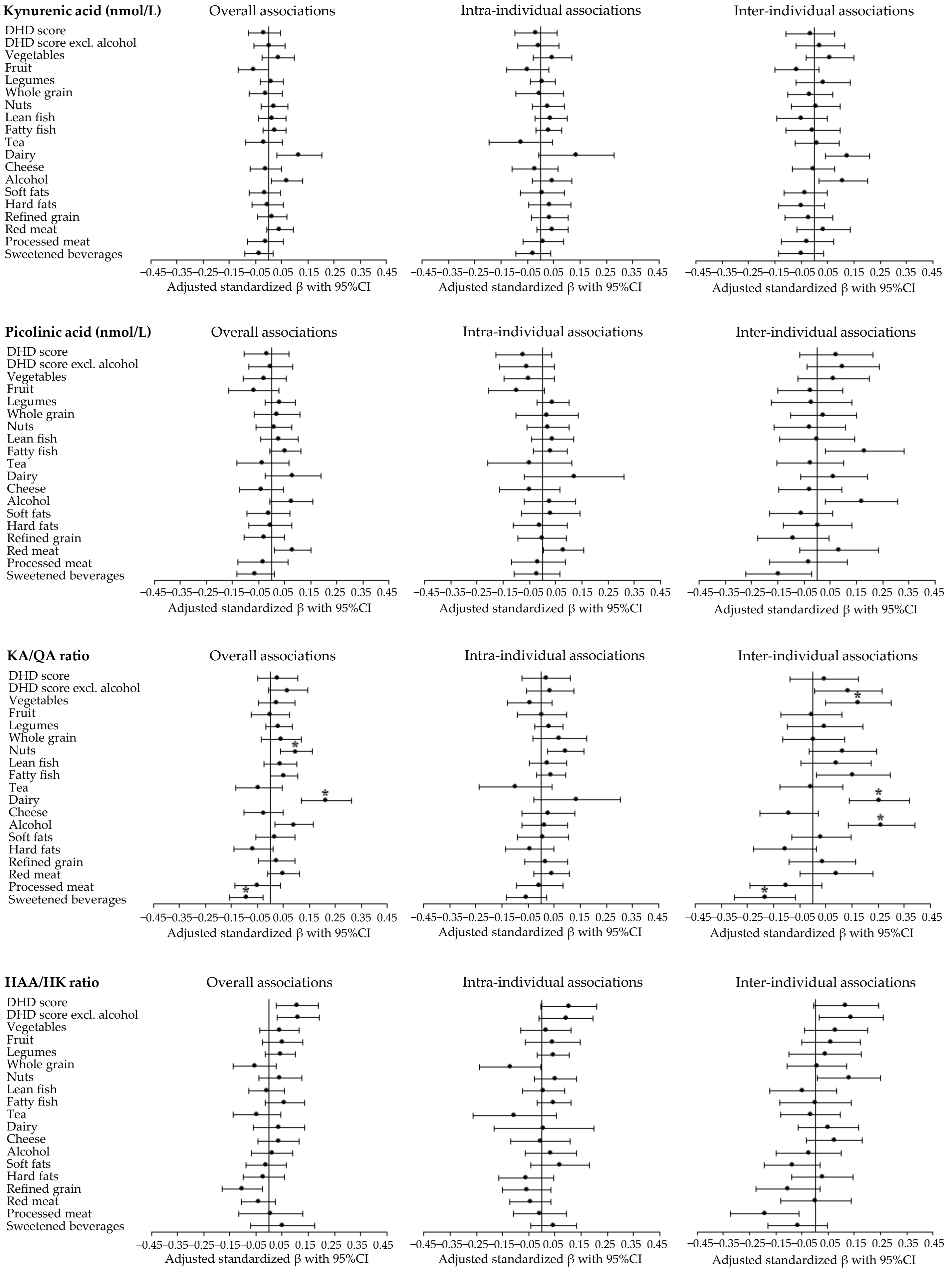

Kynurenines with less well characterized properties

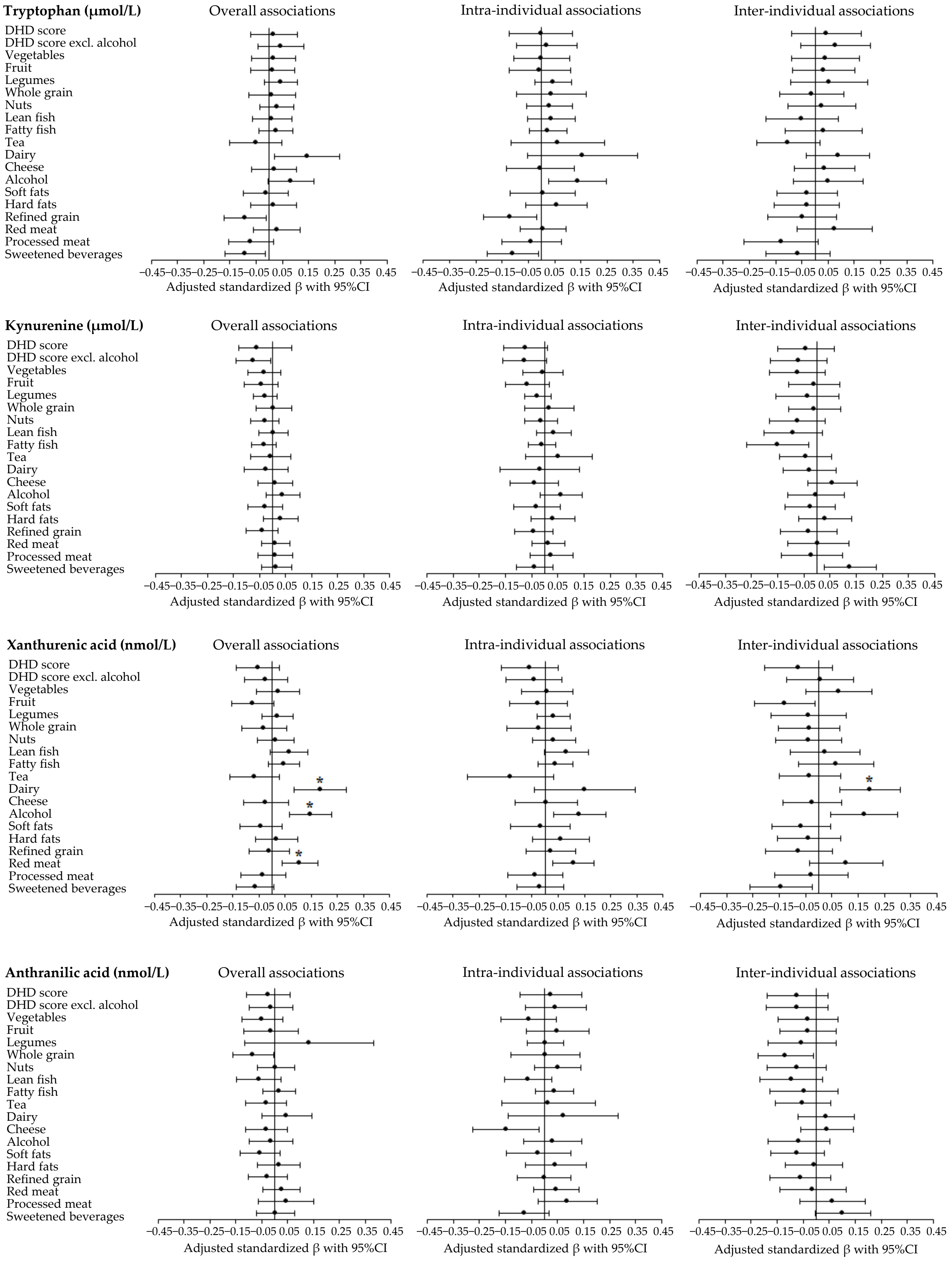

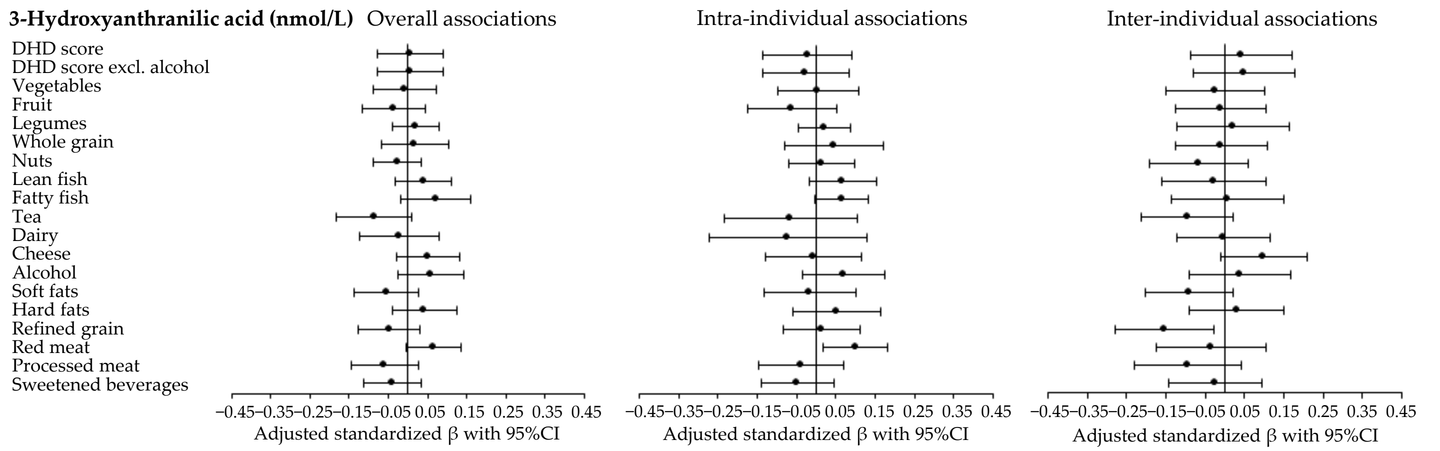

Kynurenines and ratios with neurotoxic properties

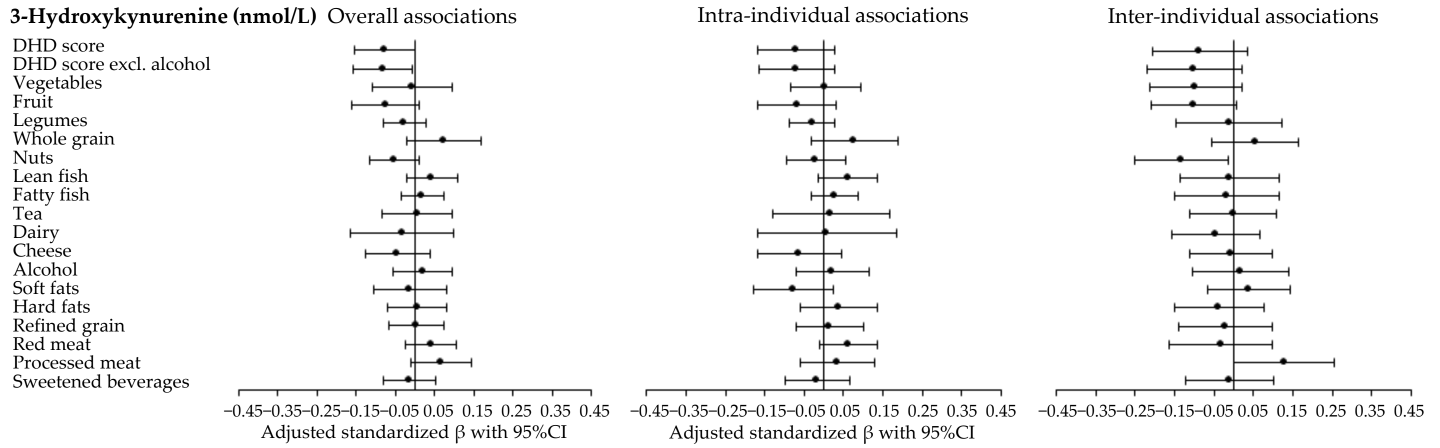

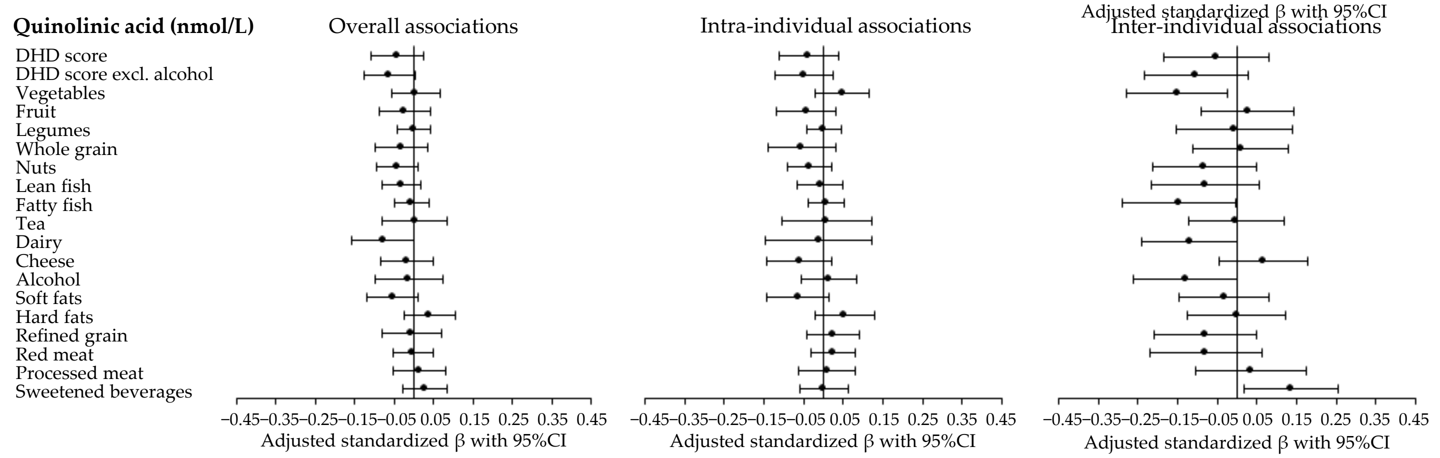

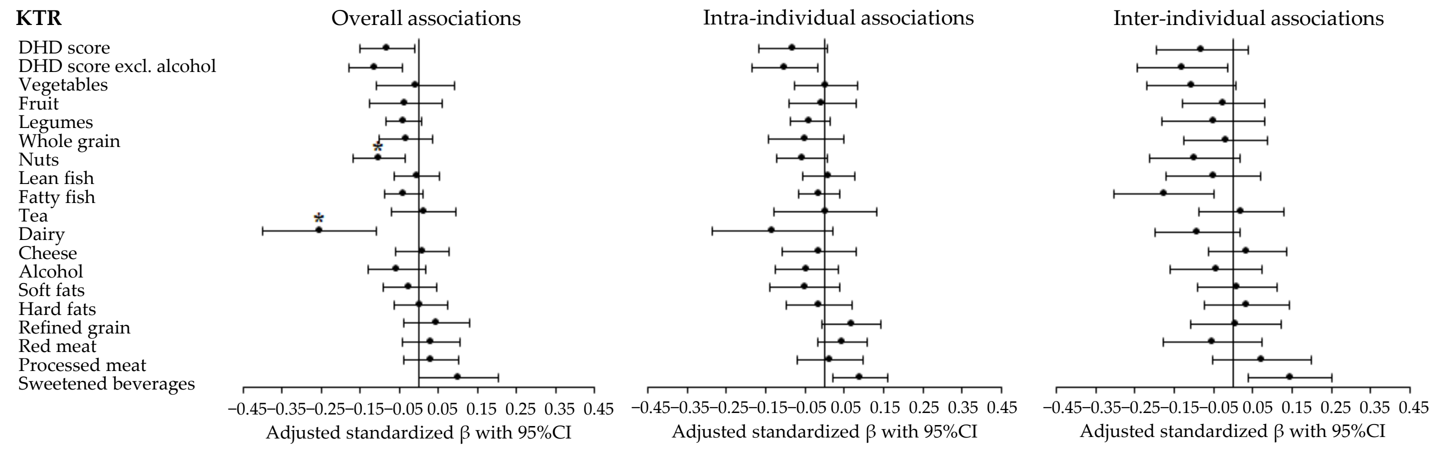

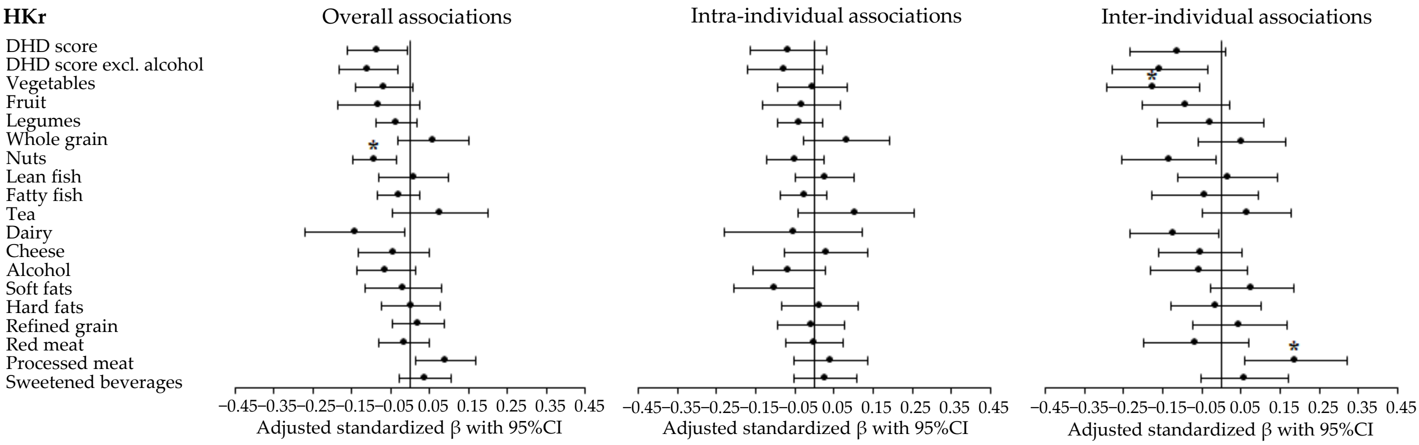

Supplementary figure S2. Forest plots of confounder-adjusted linear mixed models between adherence to the DHD recommendations and metabolites and ratios of the kynurenine pathway in colorectal cancer survivors. Analyses were adjusted for age at enrolment (years), sex (male, female), renal function (μmol/L), time since end treatment (weeks), chemotherapy (yes, no), comorbidities (0, 1, ≥2), stoma (yes, no), educational level (high, medium, low), BMI (kg/m²), total PA (hours/week), smoking status (current, former, never), and energy intake (kcal/week). A random slope was added to the model for DHD score with XA; for vegetables with HK, KA, XA, Pic, and KTR; for fruit with HK, AA, Pic, KTR, and HKr; for legumes with AA; for whole grain with HK, AA, QA, KTR, and HKr; for nuts with Trp, HAA, HAA/HK, KTR, and HKr,; for lean fish with AA, QA, and HKr; for fatty fish with HAA, and HAA/HK; for tea with AA, and HKr; for alcohol with Trp, HK, KA, QA. KTR, and HKr; for dairy with HK, XA, and HKr; for cheese with Trp, QA, and KTR; for soft fats with HK, AA, and HKr; for refined grain with AA, QA, HAA/HK, KTR, and HKr; for red meat with Trp, QA, HAA/HK, and KTR; for processed meat with Trp, KA, AA, Pic, KA/QA, and HAA/HK; and for sweetened beverages and fruit juices with HAA/HK, and KTR. The standardized β-coefficient can be interpreted as the amount of SD difference in kynurenine concentration/ratio according to 1SD difference in dietary score/intake.

* Statistically significant after FDR adjustment for multiple testing.

Abbreviations: KA, kynurenic acid; Pic, picolinic acid; KA/QA ratio, kynurenic-acid-to-quinolinic-acid ratio; HAA/HK ratio, hydroxyanthranilic-to-hydroxykynurenine ratio; Trp, tryptophan; Kyn, kynurenine; XA, xanthurenic acid; AA, anthranilic acid, HAA, 3-hydroxyanthranilic acid; HK, 3-hydroxykynurenine; QA, quinolinic acid, KTR, kynurenine-to-tryptophan ratio; HKr, hydroxykynurenine ratio.
