# Supplementary material for: Longitudinal Associations of Adherence to the Dietary World Cancer Research Fund/American Institute for Cancer Research (WCRF/AICR) and Dutch Healthy Diet (DHD) Recommendations with Plasma Kynurenines in Colorectal Cancer Survivors after Treatment
Source: Nutrients. 2022 Dec 3;14(23):5151. doi: 10.3390/nu14235151 (PMC9738623; doi:10.3390/nu14235151)
Supplement: Supplementary file 1 [file nutrients-14-05151-s001.zip › Supplementary Tables NEW.pdf]

Supplementary Table S1. Confounder-adjusted overall longitudinal, intra-individual, and inter-individual associations (*n* = 247) between the dietary WCRF/AICR recommendations and metabolites of the kynurenine pathway

		Kynurenic acid (nmol/L)	Picolinic acid (nmol/L)	Tryptophan (µmol/L)	Kynurenine (µmol/L)	Xanthurenic acid (nmol/L)	Anthranilic acid (nmol/L)	3−Hydroxy−anthranilic acid (nmol/L)	3−Hydroxy−kynurenine (nmol/L)	Quinolinic acid (nmol/L)
Dietscore	Adjusted[1,2,3]	.03 (−.05, .11)	.02 (−.09, .13)	−.01 (−.12, .11)	−.05 (−.13, .04)	.01 (−.10, .12)	−.05 (−.16, .06)	.00 (−.11, .11)	−.04 (−.13, .08)	−.06 (−.15, .02)
WCRF/AICR	Within[1,4]	.02 (−.09, .12)	−.06 (−.20, .07)	.00 (−.16, .15)	−.07 (−.17, .04)	.01 (−.13, .15)	.01 (−.15, .16)	−.01 (−.16, .13)	−.02 (−.15, .10)	−.08 (−.17, .02)
(0−5 points)	Between[1,5]	.05 (−.08, .17)	.17 (−.01, .36)	−.01 (−.19, .16)	−.01 (−.16, .13)	.00 (−.17, .18)	−.10 (−.26, .06)	.02 (−.15, .19)	.02 (−.14, .18)	−.01 (−.19, .16)
Dietscore	Adjusted[1,2,3]	.08 (−.01; .17)	.09 (−.03; .21)	.13 (−.01; .28)	−.05 (−.15; .04)	.13* (.02; .25)	−.03 (−.15; .09)	.02 (−.10; .14)	−.03 (−.17; .10)	−.12 (−.22; −.03)
WCRF/AICR excl.	Within[1,4]	.04 (−.07; .16)	−.03 (−.18; .12)	.12 (−.05; .29)	−.02 (−.15; .10)	.06 (−.09; .22)	.05 (−.12; .22)	.01 (−.15; .17)	−.01 (−.15; .10)	−.11* (−.22; .00)
alcohol (0−4 points)	Between[1,5]	.13 (−.01; .26)	.30* (.10; .49)	.13 (−.06; .32)	−.10 (−.26; .53)	.23* (.05; .42)	−.12 (−.29; .05)	.03 (−.15; .22)	−.08 (−.25; .10)	−.19 (−.37; .00)
Vegetable and fruit	Adjusted[1,2,3]	−.01 (−.08, .05)	−.06 (−.14, .02)	.02 (−.07, .11)	−.05 (−.12, .01)	−.03 (−.11, .05)	−.02 (−.13, .09)	−.03 (−.12, .05)	−.08* (−.16, −.01)	−.01 (−.08, .05)
(g/day)	Within[1,4]	.00 (−.09, .08)	−.12* (−.23, −.01)	.00 (−.12, .12)	−.05 (−.14, .03)	.00 (−.12, .11)	−.01 (−.13, .12)	−.05 (−.16, .07)	−.05 (−.15, .05)	.01 (−.07, .08)
	Between[1,5]	−.02 (−.10, .07)	.01 (−.12, .14)	.04 (−.08, .16)	−.05 (−.15, .05)	−.06 (−.18, .06)	−.04 (−.15, .07)	−.02 (−.14, .10)	−.13* (−.24, −.02)	−.06 (−.18, .06)
Dietary fibre (g/day)	Adjusted[1,2,3]	−.04 (−.11, .03)	−.05 (−.15, .05)	.03 (−.08, .14)	−.07 (−.15, .01)	−.05 (−.15, .05)	−.12* (−.22, −.02)	−.03 (−.14, .07)	−.04 (−.15, .08)	−.07 (−.15, .00)
	Within[1,4]	.03 (−.07, .12)	−.03 (−.16, .10)	.06 (−.08, .20)	−.07 (−.17, .04)	.07 (−.06, .20)	−.07 (−.21, .07)	.01 (−.13, .14)	−.01 (−.13, .11)	−.08 (−.17, .01)
	Between[1,5]	−.08 (−.18, .01)	−.07 (−.21, .07)	.00 (−.13, .14)	−.07 (−.18, .04)	−.16* (−.29, −.04)	−.15* (−.27, −.03)	−.07 (−.20, .06)	−.07 (−.19, .05)	−.05 (−.18, .08)
Alcohol (g/day)	Adjusted[1,2,3]	.12* (.03, .20)	.08 (−.02, .19)	.14* (.02, .27)	−.02 (−.11, .06)	.18* (.09, .28)	.05 (−.05, .14)	−.02 (−.12, −.08)	−.03 (−.16, .10)	−.08 (−.16, .00)
	Within[1,4]	.14 (−.01, .28)	.12 (−.07, .31)	.16 (−.05, .37)	−.02 (−.17, .13)	.15 (−.04, .35)	.07 (−.14, .28)	−.07 (−.27, .13)	.01 (−.17, .19)	−.01 (−.15, .12)
	Between[1,5]	.13* (.04, .21)	.06 (−.06, .19)	.09 (−.03, .21)	−.03 (−.13. .07)	.20* (.08, .31)	.04 (−.07, .15)	.00 (−.12, .12)	−.04 (−.16, .07)	−.12* (−.24, .00)
Ultra−processed	Adjusted[1,2,3]	−.05 (−.11, .00)	−.05 (−.12, .03)	−.04 (−.12, .04)	.03 (−.03, .09)	−.10* (−.17, −.02)	.00 (−.08, .07)	−.04 (−.12, .04)	.01 (−.06, .08)	.06 (−.01, .13)
foods (%energy)	Within[1,4]	−.01 (−.05, .03)	.02 (−.04, .08)	−.01 (−.07, .05)	.01 (−.03, .06)	−.02 (−.08, .04)	−.02 (−.08, .04)	−.02 (−.08, .04)	.00 (−.05, .06)	.03 (−.01, .07)
	Between[1,5]	−.06* (−.11, −.01)	−.11* (−.18, −.04)	−.05 (−.12, .03)	.02 (−.03, .08)	−.11* (−.17, −.04)	.01 (−.05, .08)	−.03 (−.10, .04)	.01 (−.05, .07)	.06 (−.01, .13)
Red meat	Adjusted[1,2,3]	.02 (−.04, .08)	.05 (−.04, .13)	−.07 (−.15, .02)	.01 (−.05, .08)	.05 (−.03, .14)	.09 (−.01, .19)	.00 (−.08, .09)	.09* (.00, .19)	.02 (−.05, .08)
(g/week)	Within[1,4]	.03 (−.05, .11)	.06 (−.04, .16)	−.06 (−.18, .05)	.01 (−.07, .09)	.06 (−.04, .17)	.12* (.01, .23)	.04 (−.06, .15)	.08 (−.02, .17)	.02 (−.05, .09)
	Between[1,5]	.00 (−.09, .10)	.01 (−.13, .16)	−.07 (−.21, .07)	.01 (−.10, .13)	.03 (−.11, .17)	.08 (−.04, .21)	−.07 (−.21, .07)	.12, −.01, .25)	.03 (−.11, .16)
Processed meat	Adjusted[1,2,3]	−.03 (−.09, .03)	−.04 (−.13, .05)	−.05 (−.14, .03)	.02 (−.04, .09)	−.04 (−.12, .04)	.05 (−.05, .15)	−.09* (−.18, −.01)	.01 (−.06, .09)	−.01 (−.08, .07)
(g/week)	Within[1,4]	.00 (−.08, .08)	−.06 (−.16, .04)	.00 (−.11, .12)	.01 (−.07, .09)	−.01 (−.11, .10)	.05 (−.06, .17)	−.08 (−.19, .02)	−.09 (−.18, .00)	−.05 (−.12, .02)
	Between[1,5]	−.07 (−.16, .03)	−.04 (−.18, .11)	−.14* (−.28, −.01)	.05 (−.07, .16)	−.10 (−.23, .04)	.09 (−.03, .22)	−.11 (−.24, .02)	.19* (.07, .32)	.13 (−.01, .26)
Sugar−sweetened	Adjusted[1,2]	−.04 (−.09, .02)	−.08 (−.15, .00)	−.06 (−.14, .02)	.02 (−.04, .08)	−.07 (−.14, .01)	−.01 (−.09, .07)	−.02 (−.09, .06)	−.03 (−.10, .04)	.03 (−.03, .09)
drinks (g/day)	Within[1,4]	−.02 (−.09, .05)	−.03 (−.12, .07)	.06 (−.16, .04)	−.04 (−.12, .03)	.00 (−.10, .09)	−.11* (−.21, .00)	−.03 (−.13, .07)	−.04 (−.13, .05)	−.01 (−.08, .06)
	Between[1,5]	−.06 (−.14, .02)	−.16* (−.28, −.04)	−.06 (−.18, .06)	.13* (.03, .23)	−.16* (−.27, −.04)	.09 (−.02, .20)	.00 (−.11, .12)	−.02 (−.13, 09)	.17* (.06, .29)

Abbreviations: WCRF/AICR, World Cancer Research Fund/American Institute for Cancer Research.

Both exposure and outcome variables are standardized.

Standard deviations of kynurenines are: 29 nmol/L for kynurenic acid; 15 nmol/L for picolinic acid; 12 µmol/L for tryptophan; 0.5 µmol/L for kynurenine; 8 nmol/L for xanthurenic acid; 7 nmol/L for anthranilic acid; 15 nmol/L for 3−hydroxyanthranilic acid; 27 for 3−hydroxykynurenine; and 366 nmol/L for quinolinic acid.

Standard deviations of dietary intake are: 1 point for dietscore WCRF/AICR, 128 g/day for vegetable and fruit; 6 g/day for fiber; 19 g/day for alcohol; 10% for ultra−processed foods; 301 g/week for red meat; 209 g/week for processed meat, and 132 g/day for sugar−sweetened drinks.

[1] Linear mixed models adjusted for age at enrollment (years), sex (male, female), renal function (μmol/L), time since end treatment (weeks), chemotherapy (yes, no), comorbidities (0, 1, ≥2), stoma (yes, no), educational level (high, medium, low), BMI (kg/m^2), total PA (hours/day), smoking status (current, former, never) and energy intake (kcal/week)

[2] The β-coefficients represent the overall longitudinal difference in kynurenine concentrations using linear mixed models, and can be interpreted as the amount of SD difference in kynurenine concentration/ratio according to 1SD difference in dietary score/intake

[3] A random slope was added to the model for dietscore WCRF/AICR with HK; for dietscore WCRF/AICR excl. alcohol with Trp, and HK; for vegetable and fruit with KA, and AA; for dietary fiber with HK, KA, and QA; for alcohol with Trp, HK, KA, and QA; for ultra−processed foods with QA; for red meat intake with HK, and AA; and for processed meat intake with AA, Pic, and QA

[4] The β-coefficients represent the change in kynurenine concentration over time within individuals using a hybrid model within linear mixed models

[5] The β-coefficients represent the difference in kynurenine concentration between individuals using a hybrid model within linear mixed models

* Indicates a statistically significant association

Supplementary Table S2. Confounder-adjusted overall longitudinal, intra-individual, and inter-individual associations (*n* = 247) between the dietary WCRF/AICR recommendations and relevant kynurenine ratios

		KA/QA	HAA/HK	KTR	HKr	Neopterin (nmol/L)
Dietscore	Adjusted[1,2,3]	.09 (.00, .19)	−.03 (−.15, .08)	−.08 (−.19, .03)	−.07 (−.19, .05)	.03 (−.09, .14)
WCRF/AICR	Within[1,4]	.09 (−.03, .21)	−.09 (−.23, .04)	−.11 (−.22, .00)	−.04 (−.17, .08)	.02 (−.09, .14)
(0–5 points)	Between[1,5]	.11 (−.06, .28)	.03 (−.13, .20)	.01 (−.15, .16)	−.05 (−.21, .11)	.02 (−.15, .19)
Dietscore	Adjusted[1,2,3]	.20* (.10; .31)	.01 (−.13; .14)	−.16* (−.28; −.04)	−.12 (−.26; .02)	−.06 (−.18, .05)
WCRF/AICR excl.	Within[1,4]	.12 (−.01; .25)	−.09 (−.24; .06)	−.18* (−.30; −.05)	−.06 (−.20; .08)	.00 (−.13, .13)
alcohol (0–4 points)	Between[1,5]	.36* (.17; .54)	.11 (−.07; .28)	−.18* (−.35; −.02)	−.23* (−.41; −.06)	−.21* (−.39, −.03)
Vegetable and fruit	Adjusted[1,2,3]	.01 (−.06, .09)	.06 (−.01, .14)	−.04 (−.14, .06)	−.08* (−.15, −.01)	.03 (−.05, .12)
(g/day)	Within[1,4]	−.03 (−.13, .06)	.04 (−.07, .15)	−.01 (−.10, .08)	−.03 (−.14, .07)	.05 (−.04, .14)
	Between[1,5]	.09 (−.03, .21)	.09 (−.02, .21)	−.07 (−.18, .03)	−.16* (−.27, −.04)	.03 (−.09, .14)
Dietary fiber (g/day)	Adjusted[1,2,3]	.05 (−.04, .14)	.02 (−.08, .12)	−.06 (−.14, .02)	−.01 (−.12, .09)	−.02 (−.11, .07)
	Within[1,4]	.08 (−.03, .19)	−.02 (−.14, .11)	−.05 (−.16, .05)	.01 (−.11, .13)	−.01 (−.12, .10)
	Between[1,5]	.01 (−.12, .13)	.05 (−.07, .18)	−.07 (−.18, .05)	−.04 (−.16, .08)	−.03 (−.16, .10)
Alcohol (g/day)	Adjusted[1,2,3]	.22* (.12, .31)	.04 (−.06, .14)	−.25* (−.40, −.11)	−.14* (−.27, −.01)	−.14* (−.25, −.04)
	Within[1,4]	.13 (−.03, .30)	.01 (−.18, .20)	−.13 (−.29, .02)	−.05 (−.23, .12)	−.07 (−.23, .09)
	Between[1,5]	.25* (.14, .37)	.05 (−.06, .16)	−.09 (−.20, .02)	−.12* (−.23, −.01)	−.15* (−.26, −.03)
Ultra−processed	Adjusted[1,2,3]	−.11* (−.18, −.04)	.01 (−.07, .09)	.06 (−.03, .14)	.06 (−.03, .14)	.04 (−.02, .11)
foods (%energy)	Within[1,4]	−.03 (−.08, .02)	.03 (−.03, .08)	.05* (.00, .09)	.01 (−.04, .07)	.01 (−.04, .06)
	Between[1,5]	−.14* (−.21, −.07)	−.03 (−.10, .04)	.05 (−.01, .11)	.07* (.01, .14)	.06 (−.01, .13)
Red meat (g/week)	Adjusted[1,2,3]	.00 (−.08, .07)	−.14* (−.22, −.06)	.07 (−.02, .16)	.09* (.01, .17)	.06 (−.02, .14)
	Within[1,4]	.01 (−.08, .10)	−.13* (−.22, −.03)	.06 (−.02, .14)	.06 (−.04, .15)	.08 (−.01, .16)
	Between[1,5]	−.04 (−.17, .10)	−.17* (−.30, −.04)	.08 (−.04, .21)	.15* (.02, .28)	.04 (−.10, .17)
Processed meat	Adjusted[1,2,3]	−.04 (−.12, .03)	−.02 (−.14, .10)	.02 (−.07, .11)	.07 (−.01, .14)	.05 (−.03, .13)
(g/week)	Within[1,4]	.01 (−.08, .10)	.01 (−.09, .10)	−.01 (−.09, .07)	−.05 (−.14, .05)	.01 (−.07, .10)
	Between[1,5]	−.16* (−.29, −.03)	−.25* (−.37, −.12)	.17* (.05, .29)	.28* (.16, .40)	.15 (.02, .28)
Sugar−sweetened	Adjusted[1,2,3]	−.10* (−.17, −.03)	.06 (−.07, .19)	.08 (−.01, .18)	.04 (−.02, .10)	.04 (−.05, .12)
drinks (g/day)	Within[1,4]	−.04 (−.13, .04)	.05 (−.04, .15)	.04 (−.03, .12)	.00 (−.09, .08)	−.03 (−.11, .05)
	Between[1,5]	−.21* (−.32, −.10)	−.05 (−.16, .06)	.14* (.04, .25)	.05 (−.06, .16)	.20* (.09, .31)

Abbreviations: WCRF/AICR, World Cancer Research Fund/American Institute for Cancer Research; KA/QA, kynurenic-acid-to-quinolinic-acid ratio; HAA/HK, 3-hydroxyanthranilic-acid-to-3-hydroxykynurenine ratio; KTR, kynurenine-to-tryptophan ratio; and HKr, hydroxykynurenine ratio. Both exposure and outcome variables are standardized.

Standard deviations of ratios are: 0.05 for KA/QA, 0.4 for HAA/HK; 10 for KTR; 0.2 for HKr; and 11 nmol/L for neopterin.

Standard deviations of dietary intake are: 1 point for dietscore WCRF/AICR, 128 g/day for vegetable and fruit; 6 g/day for fiber; 19 g/day for alcohol; 10% for ultra−processed foods; 301 g/week for red meat; 209 g/week for processed meat, and 132 g/day for sugar−sweetened drinks.

[1] Linear mixed models adjusted for age at enrollment (years), sex (male, female), renal function (µmol/L), time since end treatment (weeks), chemotherapy (yes, no), comorbidities (0, 1, ≥2), stoma (yes, no), educational level (high, medium, low), BMI (kg/m^2), total PA (hours/day), smoking status (current, former, never) and energy intake (kcal/week)

[2] The β-coefficients represent the overall longitudinal difference in kynurenine concentrations using linear mixed models, and can be interpreted as the amount of SD difference in kynurenine concentration/ratio according to 1SD difference in dietary score/intake

[3] A random slope was added to the model for dietscore WCRF/AICR with KTR, HKr, HAA/HK, and Neopt; for dietscore WCRF/AICR excl alcohol with KTR, HKr, and HAA/HK; for vegetable and fruit with KTR, HKr, and Neopt; for dietary fiber with HKr; for alcohol with KTR, HKr, and Neopt; for ultra−processed foods with KTR, HKr, and HAA/HK; for red meat with KTR, and Neopt; for processed meat with KTR, HAA/HK, and Neopt; and for sugar−sweetened drink intake with KTR, HKr, HAA/HK, and Neopt.

[4] The β-coefficients represent the change in kynurenine ratios over time within individuals using a hybrid model within linear mixed models

[5] The β-coefficients represent the difference in kynurenine ratios between individuals using a hybrid model within linear mixed models

* Indicates a statistically significant association

Supplementary Table S3. Confounder-adjusted overall longitudinal, intra-individual, and inter-individual associations (*n* = 247) between the DHD recommendations and metabolites of the kynurenine pathway

		Kynurenic acid (nmol/L)	Picolinic acid (nmol/L)	Tryptophan (μmol/L)	Kynurenine (μmol/L)	Xanthurenic acid (nmol/L)	Anthranilic acid (nmol/L)	3−Hydroxy−anthranilic acid (nmol/L)	3−Hydroxy−Kynurenine (nmol/L)	Quinolinic acid (nmol/L)
Total DHD score (0−130 points)	Adjusted[1,2]	−.02 (−.08; .04)	−.02 (−.10; .07)	.02 (−.07; .11)	−.06 (−.13; .01)	−.05 (−.14; .03)	−.02 (−.11; .06)	.01 (−.08; .09)	−.08 (−.15; .00)	−.04 (−.11; .02)
	Within[1,4]	−.02 (−.10; .06)	−.07 (−.18; .03)	.00 (−.12; .12)	−.07 (−.16; .01)	−.06 (−.17; .05)	.02 (−.09; .14)	−.02 (−.13; .09)	−.07 (−.17; .03)	−.04 (−.11; .04)
	Between[1,5]	−.02 (−.11; .08)	.07 (−.07; .21)	.04 (−.09; .18)	−.04 (−.15; .07)	−.08 (−.21; .05)	−.07 (−.19; .05)	.04 (−.09; .17)	−.09 (−.21; .04)	−.05 (−.18; .08)
Total DHD score excl. alcohol (0−120 points)	Adjusted[1,2]	.00 (−.06; .06)	.00 (−.09; .08)	.05 (−.04; .13)	−.07* (−.14; −.01)	−.02 (−.11; .06)	−.01 (−.10; .07)	.01 (−.08; .09)	−.08* (−.16; −.01)	−.06 (−.13; .00)
	Within[1,4]	−.01 (−.09; .07)	−.06 (−.16; .04)	.02 (−.10; .14)	−.08 (−.16; .01)	−.04 (−.15; .06)	.04 (−.07; .16)	−.03 (−.14; .08)	−.07 (−.16; .03)	−.05 (−.12; .03)
	Between[1,5]	.02 (−.07; .11)	.10 (−.04; .24)	.08 (−.05; .21)	−.07 (−.18; .04)	.01 (−.12; .13)	−.07 (−.19; .04)	.05 (−.08; .18)	−.10 (−.22; .02)	−.10 (−23; .03)
Vegetables (g/day)	Adjusted[1,2,3]	.04 (−.02; .10)	−.03 (−.11; .06)	.02 (−.07; .10)	−.03 (−.09; .03)	.02 (−.06; .11)	−.05 (−.13; .03)	−.01 (−.09; .07)	−.01 (−.11; .10)	.00 (−.06; .07)
	Within[1,4]	.05 (−.03; .12)	−.05 (−.15; .04)	.00 (−.11; .11)	−.01 (−.08; .07)	.01 (−.09; .11)	−.06 (−.17; .05)	.00 (−.10; .11)	.00 (−.09; .09)	.05 (−.02; .12)
	Between[1,5]	.06 (−.03; .15)	.06 (−.07; .20)	.04 (−.09; .17)	−.07 (−.18; .03)	.08 (−.05; .20)	−.03 (−.15; .08)	−.03 (−.15; .10)	−.10 (−.21; .02)	−.15* (−.28; −.02)
Fruit (g/day)	Adjusted[1,2,3]	−.06 (−.12; .00)	−.07 (−.16; .03)	.01 (−.07; .10)	−.04 (−.11; .02)	−.07 (−.15; .01)	−.01 (−.12; .09)	−.04 (−.12; .05)	−.07 (−.16; .01)	−.02 (−.09; .04)
	Within[1,4]	−.05 (−.13; .03)	−.10 (−.20; .01)	−.01 (−.12; .11)	−.07 (−.15; .02)	−.03 (−.13; .08)	.05 (−.07; .17)	−.06 (−.17; .05)	−.07 (−.17; .03)	−.04 (−.12; .03)
	Between[1,5]	−.07 (−.15; .02)	−.03 (−.15; .10)	.03 (−.09; .15)	−.01 (−.11; .09)	−.13* (−.24; −.01)	−.03 (−.14; .08)	−.01 (−.13; .11)	−.10 (−.21; .01)	.03 (−.09; .14)
Legumes (g/day)	Adjusted[1,2]	.01 (−.03; .06)	.03 (−.03; .09)	.05 (−.02; .11)	−.03 (−.07; .02)	.02 (−.04; .08)	.13 (−.12; .38)	.02 (−.04; .08)	−.03 (−.08; .03)	.00 (−.04; .04)
	Within[1,4]	.01 (−.04; .06)	.04 (−.02; .10)	.04 (−.03; .11)	−.03 (−.08; .02)	.03 (−.03; .10)	.01 (−.06; .08)	.02 (−.04; .09)	−.03 (−.09; .03)	.00 (−.04; .04)
	Between[1,5]	.03 (−.07; .14)	−.02 (−.17; .13)	.06 (−.09; .20)	−.04 (−.16; .08)	−.04 (−.18; .11)	−.06 (−.19; .08)	.02 (−.12; .16)	−.01 (−.15; .12)	−.01 (−.15; .14)
Whole grain (g/day)	Adjusted[1,2,3]	−.01 (−.07; .05)	.02 (−.07; .11)	.01 (−.08; .10)	.00 (−.06; .07)	−.03 (−.11; .06)	−.08* (−.16; .00)	.02 (−.07; .10)	.07 (−.02; .17)	−.03 (−.10; .03)
	Within[1,4]	.00 (−.09; .09)	.02 (−.10; .14)	.04 (−.09; .17)	.02 (−.08; .11)	−.02 (−.14; .10)	.00 (−.13; .14)	.05 (−.08; .17)	.08 (−.03; .19)	−.05 (−.14; .03)
	Between[1,5]	−.02 (−.10; .07)	.02 (−.10; .15)	−.01 (−.13; .11)	−.01 (−.11; .09)	−.04 (−.15; .08)	−.12* (−.22; −.01)	−.01 (−.13; .11)	.05 (−.06; .17)	.01 (−.11; .13)
Nuts (g/day)	Adjusted[1,2,3]	.02 (−.03; .07)	.01 (−.06; .08)	.03 (−.03; .09)	−.03 (−.08; .03)	.01 (−.06; .08)	.00 (−.07; .08)	−.03 (−.09; .03)	−.05 (−.11; .01)	−.04 (−.09; .01)
	Within[1,4]	.03 (−.03; .09)	.02 (−.06; .10)	.03 (−.06; .12)	−.01 (−.08; .05)	.03 (−.05; .12)	.05 (−.04; .14)	.01 (−.07; .10)	−.02 (−.09; .05)	−.03 (−.09; .02)
	Between[1,5]	.01 (−.09; .10)	−.03 (−.17; .11)	.03 (−.10; .16)	−.07 (−.18; .03)	−.04 (−.16; .09)	−.07 (−.19; .04)	−.07 (−.19; .06)	−.13* (−.25; −.02)	−.08 (−.21; .05)
Lean fish (g/day)	Adjusted[1,2,3]	.01 (−.04; .07)	.03 (−.04; .10)	.01 (−.07; .09)	.00 (−.05; .06)	.07 (−.01; .14)	−.06 (−.15; .03)	.04 (−.03; .11)	.04 (−.02; .11)	−.03 (−.08; .02)
	Within[1,4]	.04 (−.02; .10)	.04 (−.04; .12)	.04 (−.05; .13)	.04 (−.03; .10)	.08 (.00; .16)	−.06 (−.15; .03)	.07 (−.02, .15)	.06 (−.01; .14)	−.01 (−.07; .05)
	Between[1,5]	−.05 (−.14; .05)	.00 (−.14; .14)	−.05 (−.19; .09)	−.09 (−.20; .02)	.02 (−.11; .16)	−.10 (−.22; .03)	−.03 (−.16; .11)	−.01 (−.14; .11)	−.08 (−.22; .06)
Fatty fish (g/day)	Adjusted[1,2]	.02 (−.02; .07)	.05 (−.01; .11)	.03 (−.04; .09)	−.03 (−.08; .02)	.04 (−.01; .10)	.02 (−.04; .08)	.07 (−.02; .16)	.02 (−.02; .07)	−.01 (−.05; .04)
	Within[1,4]	.03 (−.02; .08)	.03 (−.03; .09)	.02 (−.05; .10)	−.01 (−.06; .04)	.04 (−.03; .11)	.04 (−.03; .11)	.07 (.00; .13)	.03 (−.03; .09)	.01 (−.04; .05)
	Between[1,5]	−.01 (−.11; .10)	.18 (.03; .33)*	.03 (−.11; .18)	−.15* (−.27; −.03)	.07 (−.08; .21)	−.05 (−.18; .08)	.01 (−.14; .15)	−.02 (−.15; .12)	−.15* (−.29; .00)
Tea (g/day)	Adjusted[1,2,3]	−.02 (−.09; .05)	−.03 (−.13; .07)	−.05 (−.15; .05)	−.01 (−.09; .07)	−.07 (−.16; .03)	−.03 (−.11; .05)	−.09 (−.18; .01)	.01 (−.08; .09)	.00 (−.08; .09)
	Within[1,4]	−.07 (−.20; .05)	−.05 (−.21; .11)	.06 (−.12; .50)	.05 (−.08; .18)	−.13 (−.30; .03)	.02 (−.16; .19)	−.07 (−.24; .10)	.02 (−.13; .17)	.01 (−.11; .12)
	Between[1,5]	.01 (−.07; .10)	−.02 (−.15; .10)	−.10 (−.22; .02)	−.04 (−.14; .06)	−.03 (−.15; .08)	−.05 (−.16; .06)	−.10 (−.21; .02)	−.00 (−.11; .11)	.00 (−.12; .12)
Alcohol (g/day)	Adjusted[1,2,3]	.12 (.03; .20)	.08 (−.02; .19)	.14 (.02; .27)*	−.02 (−.11; .06)	.18* (.09; .28)	.05 (−.05; .14)	−.02 (−.12; .08)	−.03 (−.16; .10)	−.08 (−.16; .00)
	Within[1,4]	.14 (−.01; .28)	.12 (−.07; .31)	.16 (−.05; .37)	−.02 (−.17; .13)	.15 (−.04; .35)	.07 (−.14; .28)	−.07 (−.27; .13)	.01 (−.17; .18)	−.01 (−.15; .12)
	Between[1,5]	.13 (.04; .21)	.06 (−.06; .19)	.09 (−.03; .21)	−.03 (−.13; .07)	.20* (.08; .31)	.04 (−.07; .15)	.00 (−.12; .12)	−.04 (−.16; .07)	−.12 (−.24; .00)
Dairy (g/day)	Adjusted[1,2,3]	−.01 (−.07; .05)	−.04 (−.12; .05)	.02 (−.07; .10)	.01 (−.06; .08)	−.02 (−.11; .06)	−.03 (−.11; .05)	.05 (−.03; .13)	−.04 (−.13; .04)	−.02 (−.08; .05)

	Within[1,4]	−.02 (−.11; .07)	−.05 (−.16; .07)	.00 (−.13; .12)	−.04 (−.13; .05)	.01 (−.11; .12)	−.15* (−.27; −.02)	−.01 (−.13; .12)	−.06 (−.17; .04)	−.06 (−.14; .02)
	Between[1,5]	.00 (−.08; .08)	−.03 (−.15; .09)	.04 (−.08; .15)	.06 (−.03; .15)	−.02 (−.14; .09)	.04 (−.06; .14)	.10 (−.01; .21)	−.01 (−.11; .10)	.07 (−.05; .18)
Cheese (g/day)	Adjusted[1,2]	.07* (.01; .13)	.08 (−.01; .16)	.08 (.00; .17)	.04 (−.03; .11)	.15* (.07; .23)	−.01 (−.10; .07)	.06 (−.02; .14)	.02 (−.06; .09)	−.01 (−.10; .07)
	Within[1,4]	.04 (−.03; .12)	.03 (−.07; .13)	.14* (.03; .25)	.06 (−.02; .14)	.13* (.03; .23)	.03 (−.08; .14)	.07 (−.03; .18)	.02 (−.07; .11)	.01 (−.06; .09)
	Between[1,5]	.11* (.02; .20)	.17* (.03; .31)	.05 (−.08; .18)	.00 (−.11; .11)	.17* (.05; .30)	−.07 (−.18; .05)	.04 (−.09; .17)	.02 (−.10; .14)	−.13 (−.26; .00)
Soft fats (g/day)	Adjusted[1,2,3]	−.01 (−.07; .05)	−.01 (−.09; .07)	−.01 (−.10; .07)	−.03 (−.09; .04)	−.04 (−.12; .04)	−.05 (−.13; .02)	−.06 (−.14; .03)	−.01 (−.11; .08)	−.05 (−.12; .01)
	Within[1,4]	.01 (−.08; .09)	.03 (−.08; .14)	.01 (−.12; .13)	−.03 (−.12; .06)	−.02 (−.13; .10)	−.02 (−.15; .10)	−.02 (−.13; .10)	−.08 (−.18; .03)	−.06 (−.14; .02)
	Between[1,5]	−.03 (−.11; .05)	−.06 (−.18; .06)	−.03 (−.14; .09)	−.03 (−.12; .07)	−.07 (−.18; .05)	−.07 (−.18; .03)	−.09 (−.20; .02)	.04 (−.07; .14)	−.03 (−.15; .08)
Hard fats (g/day)	Adjusted[1,2]	.00 (−.06; .06)	.00 (−.09; .08)	.02 (−.07; .10)	.03 (−.03; .10)	.02 (−.06; .10)	.02 (−.07; .10)	.04 (−.04; .13)	.01 (−.07; .08)	.04 (−.02; .10)
	Within[1,4]	.03 (−.04; .11)	−.01 (−.11; .10)	.06 (−.06; .18)	.03 (−.05; .12)	.06 (−.05; .17)	.04 (−.07; .16)	.05 (−.06; .16)	.04 (−.06; .13)	.05 (−.02; .13)
	Between[1,5]	−.05 (−.14; .04)	.00 (−.11; .10)	−.03 (−.16; .09)	.03 (−.07; .13)	−.04 (−.16; .08)	−.01 (−.12; .10)	.03 (−.09; .15)	−.04 (−.15; .08)	.00 (−.12; .12)
Refined grain	Adjusted[1,2,3]	.01 (−.04; .07)	−.03 (−.11; .05)	−.09* (−.17; −.01)	−.04 (−.10; .02)	−.01 (−.09; .07)	−.03 (−.10; .05)	−.05 (−.13; .03)	.00 (−.07; .07)	−.01 (−.08; .07)
	Within[1,4]	.03 (−.04; .10)	.00 (−.09; .09)	−.12* (−.22; −.02)	−.04 (−.12; .03)	.02 (−.07; .12)	.00 (−.10; .10)	.01 (−.08; .11)	.01 (−.07; .10)	.02 (−.04; .09)
	Between[1,5]	−.02 (−.11; .07)	−.09 (−.23; .05)	−.05 (−.18; .08)	−.03 (−.14; .08)	−..08 (−.20; .05)	−.06 (−.18; .06)	−.15* (−.28; −.03)	−.02 (−.14; .10)	−.08 (−.21; .05)
Red meat (g/day)	Adjusted[1,2]	.04 (−.01; .09)	.08* (.01; .15)	.03 (−.06; .12)	.01 (−.04; .07)	.11* (.04; .18)	.03 (−.05; .10)	.07 (−.01; .14)	.04 (−.02; .10)	.00 (−.05; .05)
	Within[1,4]	.05 (−.01; .10)	.08* (.00; .16)	.01 (−.08; .09)	.01 (−.05; .08)	.11* (.03; .19)	.05 (−.04; .13)	.10* (.02; .18)	.06 (−.01; .14)	.02 (−.03; .08)
	Between[1,5]	.03 (−.07; .13)	.08 (−.07; .23)	.08 (−.07; .22)	.00 (−.11; .12)	.10 (−.04; .24)	−.01 (−.14; .12)	−.03 (−.17; .11)	−.03 (−.16; .10)	−.08 (−.22; .06)
Processed meat (g/day)	Adjusted[1,2,3]	−.01 (−.08; .06)	−.03 (−.13; .06)	−.07 (−.16; .02)	.01 (−.06; .08)	−.03 (−.12; .05)	.05 (−.06; .15)	−.06 (−.15; .03)	.07 (−.01; .14)	.01 (−.05; .08)
	Within[1,4]	.01 (−.07; .09)	−.02 (−.12; .09)	−.04 (−.15; .08)	.02 (−.06; .11)	−.04 (−.14; .07)	.09 (−.03; .20)	−.04 (−.15; .07)	.03 (−.06; .13)	.01 (−.06; .08)
	Between[1,5]	−.03 (−.13; .07)	−.03 (−.18; .12)	−.13 (−.27; .01)	−.02 (−.14; .10)	−.03 (−.17; .11)	.06 (−.06; .19)	−.09 (−.23; .04)	.13 (.00; .26)	.03 (−.11; .17)
Sweetened beverages and fruit juices (g/day)	Adjusted[1,2,3]	−.04 (−.09; .02)	−.06 (−.13; .01)	−.09* (−.17; −.02)	.02 (−.04; .07)	−.06 (−.14; .01)	.00 (−.07; .08)	−.04 (−.11; .04)	−.01 (−.08; .05)	.03 (−.03; .08)
	Within[1,4]	−.03 (−.09; .04)	−.02 (−.11; .06)	−.11* (−.21; −.01)	−.04 (−.11; .03)	−.02 (−.11; .07)	−.08 (−.18; .02)	−.05 (−.14; .04)	−.02 (−.10; .06)	.00 (−.06; .06)
	Between[1,5]	−.05 (−.14; .03)	−.15 (−.27; −.02)	−.06 (−.19; .06)	.13* (.03; .23)	−.14* (−.26; −.03)	.10 (.00; .21)	−.02 (−.14; .09)	−.01 (−.12; .10)	.14* (.02; .25)

Abbreviations: DHD, Dutch Healthy Diet score.

Both exposure and outcome variables are standardized.

Standard deviations of kynurenines are: 29 nmol/L for kynurenic acid; 15 nmol/L for picolinic acid; 12 μmol/L for tryptophan; 0.5 μmol/L for kynurenine; 8 nmol/L for xanthurenic acid; 7 nmol/L for anthranilic acid; 15 nmol/L for 3−hydroxyanthranilic acid; 27 for 3−hydroxykynurenine; and 366 nmol/L for quinolinic acid.

Standard deviations of dietary intake are: 15 points for DHD score; 71 g/day for vegetables; 90 g/day for fruit; 10 g/day for legumes; 63 g/day for whole grain; 7 g/day for nuts; 16 g/day for lean fish; 15 g/day for fatty fish; 264 g/day for tea; 19 g/day for alcohol; 143 g/day for dairy; 21 g/day for cheese; 14 g/day for soft fats; 12 g/day for hard fats; 51 g/day for refined grain; 27 g/day for red meat; 33 g/day for processed meat; and 115 g/day for sweetened beverages and fruit juices.

[1] Linear mixed models adjusted for age at enrollment (years), sex (male, female), renal function (μmol/L), time since end treatment (weeks), chemotherapy (yes, no), comorbidities (0, 1, ≥2), stoma (yes, no), educational level (high, medium, low), BMI (kg/m^2), total PA (hours/day), smoking status (current, former, never) and energy intake (kcal/week)

[2] The β-coefficients represent the overall longitudinal difference in kynurenine concentrations using linear mixed models, and can be interpreted as the amount of SD difference in kynurenine concentration/ratio according to 1SD difference in dietary score/intake

[3] A random slope was added to the model for DHD score with XA; for vegetables with HK, KA, XA, and Pic; for fruit with HK, AA, and Pic; for legumes with AA; for whole grain with HK, AA, and QA; for nuts with Trp, and HAA; for lean fish with AA, and QA; for fatty fish with HAA; for tea with AA; for alcohol with Trp, HK, KA, and QA; for dairy with HK, and XA; for cheese with Trp, and QA; for soft fats with HK, and AA; for refined grain with AA, and QA; for red meat with Trp, and QA; and for processed meat with Trp, KA, AA, and Pic

[4] The β-coefficients represent the change in kynurenine concentration over time within individuals using a hybrid model within linear mixed models

[5] The β-coefficients represent the difference in kynurenine concentration between individuals using a hybrid model within linear mixed models

* Indicates a statistically significant association

Supplementary Table S4. Confounder-adjusted overall longitudinal, intra-individual, and inter-individual associations (*n* = 247) between the DHD recommendations and relevant kynurenine ratios

		KA/QA	HAA/HK	KTR	HKr	Neopt (nmol/L)
Total DHD score	Adjusted[1,2]	.03 (−.05; .10)	.11* (.03; .19)	−.08* (−.15; −.01)	−.08* (−.16; −.01)	−.03 (−.10, .05)
(0−130 points)	Within[1,4]	.02 (−.07; .11)	.10 (.00; .21)	−.08 (−.17; .01)	−.07 (−.16; .03)	−.03 (−.12, .06)
	Between[1,5]	.04 (−.09; .18)	.12 (−.01; .24)	−.08 (−.20; .04)	−.11 (−.24; .01)	−.02 (−.15, .10)
Total DHD score	Adjusted[1,2]	.07 (−.01; .14)	.11* (.03; .19)	−.11* (−.18; −.04)	−.11* (−.18; −.03)	−.06 (−.13, .01)
excl. alcohol	Within[1,4]	.03 (−.06; .13)	.09 (−.01; .20)	−.10* (−.19; −.02)	−.08 (−.17; 02)	−.04 (−.13, .05)
(0−120 points)	Between[1,5]	.14 (.01; .26)	.14* (.02; .26)	−.13* (−.25; −.01)	−.16* (−.28; −.04)	−.08 (−.21, .04)
Vegetables	Adjusted[1,2,3]	.02 (−.05; .10)	.04 (−.04; .12)	−.01 (−.11; .09)	−.07 (−.14; .01)	.03 (−.04, .10)
(g/day)	Within[1,4]	−.04 (−.13; .04)	.02 (−.08; .11)	.00 (−.08; .08)	.00 (−.09; .09)	.06 (−.02, .14)
	Between[1,5]	.17* (.05; .30)	.08 (−.04; .20)	−.11 (−.22; .01)	−.18* (−.29; −.06)	−.04 (−.17, .08)
Fruit (g/day)	Adjusted[1,2,3]	.00 (−.07; .07)	.05 (−.03; .13)	−.03 (−.13; .06)	−.08 (−.18; .02)	.03 (−.05, .10)
	Within[1,4]	.00 (−.09; .10)	.04 (−.07; .15)	−.01 (−.09; .08)	−.03 (−.13; .07)	.00 (−.09, .09)
	Between[1,5]	.00 (−.12; .11)	.06 (−.05; .17)	−.02 (−.13; .08)	−.09 (−.20; .02)	.07 (−.05, .18)
Legumes (g/day)	Adjusted[1,2]	.03 (−.02; .08)	.04 (−.01; .10)	−.04 (−.09; .01)	−.04 (−.09; .02)	−.02 (−.06, .03)
	Within[1,4]	.03 (−.02; .09)	.04 (−.02; .11)	−.04 (−.09; .01)	−.04 (−.09; .02)	−.02 (−.07, .04)
	Between[1,5]	.05 (−.10; .19)	.04 (−.10; .18)	−.05 (−.18; .08)	−.03 (−.16; .11)	.00 (−.15, .14)
Whole grain	Adjusted[1,2,3]	.04 (−.04; .12)	−.05 (−.14; .03)	−.03 (−.10; .04)	.06 (−.03; .15)	−.04 (−.12, .04)
(g/day)	Within[1,4]	.07 (−.03; .18)	−.12* (−.24; .00)	−.05 (−.14; .05)	.08 (−.03; .19)	−.03 (−.13, .08)
	Between[1,5]	.00 (−.11; .12)	.01 (−.11; .12)	−.02 (−.13; .09)	.05 (−.06; .17)	−.06 (−.18, .06)
Nuts (g/day)	Adjusted[1,2,3]	.10* (.04; .16)	.04 (−.04; .13)	−.10* (−.17; −.03)	−.09* (−.15; −.04)	−.05 (−.10, .00)
	Within[1,4]	.09* (.02; .16)	.05 (−.03; .13)	−.06 (−.12; .01)	−.05 (−.12; .03)	−.03 (−.09, .04)
	Between[1,5]	.11 (−.01; .24)	.13* (.01; .25)	−.10 (−.21; .02)	−.13* (−.25; −.01)	−.05 (−.18, .07)
Lean fish (g/day)	Adjusted[1,2,3]	.04 (−.02; .10)	−.01 (−.08; .06)	.00 (−.06; .05)	.01 (−.08; .10)	.01 (−.06, .08)
	Within[1,4]	.03 (−.05; .10)	.01 (−.07; .09)	.01 (−.06; .08)	.03 (−.05; .10)	−.01 (−.07, .06)
	Between[1,5]	.09 (−.05; .22)	−.05 (−.17; .08)	−.05 (−.17; .07)	.02 (−.11; .14)	.06 (−.08, .19)
Fatty fish (g/day)	Adjusted[1,2]	.05* (.00; .11)	.06 (−.01; .13)	−.04 (−.09; .01)	−.03 (−.08; .03)	−.01 (−.06, .04)
	Within[1,4]	.04 (−.02; .09)	.05 (−.02; .11)	−.01 (−.07; .04)	−.03 (−.09; .03)	.00 (−.06, .05)
	Between[1,5]	.15* (.01; .30)	.00 (−.14; .14)	−.18* (−.30; −.05)	−.04 (−.18; .09)	−.10 (−.24, .04)
Tea (g/day)	Adjusted[1,2,3]	−.04 (−.13; .05)	−.05 (−.14; .05)	.01 (−.07; .10)	.08 (−.04; .20)	.03 (−.06, .12)
	Within[1,4]	−.10 (−.24; .04)	−.11 (−.26; .05)	.00 (−.13; .13)	.11 (−.04; .25)	−.02 (−.16, .11)
	Between[1,5]	−.01 (−.12; .11)	−.02 (−.13; .10)	.02 (−.09; .13)	.07 (−.05; .18)	.07 (−.04, .19)
Alcohol (g/day)	Adjusted[1,2,3]	.22* (.12; .31)	.04 (−.06; .14)	−.25* (−.40; −.11)	−.14* (−.27; −.01)	−.14* (−.25, −.04)
	Within[1,4]	.14 (−.03; .30)	.01 (−.18; .20)	−.13 (−.29; .02)	−.05 (−.23; .12)	−.07 (−.23, .09)
	Between[1,5]	.25* (.14; .37)	.05 (−.06; .16)	−.09 (−.20; .02)	−.12* (−.23; −.01)	−.15* (−.26, −.03)
Dairy (g/day)	Adjusted[1,2,3]	−.02 (−.10; .05)	.04 (−.04; .12)	.01 (−.06; .08)	−.04 (−.13; .05)	−.04 (−.11, .03)
	Within[1,4]	.03 (−.07; .13)	−.01 (−.12; .11)	−.01 (−.11; .08)	.03 (−.08; .14)	−.06 (−.16, .04)
	Between[1,5]	−.09 (−.20; .02)	.07 (−.03; .18)	.04 (−.06; .14)	−.05 (−.16; .05)	−.02 (−.13, .09)
Cheese (g/day)	Adjusted[1,2]	.09* (.02; .17)	.01 (−.07; .09)	−.06 (−.13; .02)	−.06 (−.14; .01)	−.04 (−.11, .04)
	Within[1,4]	.01 (−.07; .10)	.03 (−.06; .13)	−.04 (−.13; .04)	−.06 (−.16; .03)	−.01 (−.09, .08)
	Between[1,5]	.26* (.14; .39)	−.02 (−.15; .10)	−.04 (−.16; .07)	−.06 (−.18; .07)	−.10 (−.23, .02)
Soft fats (g/day)	Adjusted[1,2,3]	.02 (−.06; .09)	−.01 (−.09; .07)	−.02 (−.09; .04)	−.02 (−.12; .08)	−.04 (−.13, .06)
	Within[1,4]	.01 (−.09; .11)	.07 (−.04; .18)	−.05 (−.14; .04)	−.10 (−.20; .00)	−.07 (−.16, .02)
	Between[1,5]	.03 (−.08; .15)	−.09 (−.19; .02)	.01 (−.09; .11)	.08 (−.03; .18)	.03 (−.08, .14)
Hard fats (g/day)	Adjusted[1,2]	−.07 (−.14; .01)	−.02 (−.10; .06)	.00 (−.06; .07)	.00 (−.07; .08)	.03 (−.04, .10)
	Within[1,4]	−.04 (−.13; .05)	−.06 (−.16; .04)	−.01 (−.10; .07)	.01 (−.08; .11)	.04 (−.05, .13)
	Between[1,5]	−.11 (−.23; .02)	.03 (−.09; .15)	.04 (−.07; .14)	−.01 (−.13; .10)	.01 (−.11, .13)
Refined grain	Adjusted[1,2,3]	.02 (−.04; .09)	−.10* (−.18; −.03)	.05 (−.04; .13)	.02 (−.05; .09)	.03 (−.03, .10)
	Within[1,4]	.02 (−.06; .10)	−.06 (−.15; .03)	.07 (−.01; .14)	−.01 (−.09; .08)	.02 (−.05, .10)
	Between[1,5]	.04 (−.09; .17)	−.10 (−.23; .02)	.01 (−.11; .12)	.05 (−.07; .17)	.06 (−.07, .18)
Red meat (g/day)	Adjusted[1,2,3]	.05 (−.01; .11)	−.04 (−.11; .02)	.03 (−.04; .11)	−.02 (−.08; .05)	.03 (−.03, .09)
	Within[1,4]	.04 (−.03; .11)	−.04 (−.12; .04)	.05 (−.02; .11)	.00 (−.07; .07)	.06 (−.01, .13)
	Between[1,5]	.09 (−.05; .23)	.00 (−.13; .14)	−.05 (−.18; .07)	−.06 (−.20; .07)	−.08 (−.22, .06)
Processed meat	Adjusted[1,2,3]	−.05 (−.14; .04)	.01 (−.12; .13)	.03 (−.04; .10)	.09* (.01; .17)	.05 (−.03, .13)
(g/day)	Within[1,4]	−.01 (−.10; .08)	−.01 (−.11; .09)	.01 (−.07; .10)	.04 (−.05; .13)	.03 (−.05, .12)
	Between[1,5]	−.10 (−.24; .04)	−.19* (−.32; −.06)	.07 (−.05; .20)	.19* (.06; .32)	.07 (−.07, .20)
	Adjusted[1,2,3]	−.09* (−.16; −.03)	.05 (−.07; .18)	.10 (.00; .20)	.04 (−.03; .11)	.07 (−.02, .16)
	Within[1,4]	−.06 (−.13; .02)	.05 (−.04; .13)	.09* (.02; .16)	.03 (−.05; .11)	.02 (−.05, .10)

Sweetened beverages and fruit juices (g/day)	Between[1,5]	−.18* (−.30; −.06)	−.07 (−.18; .05)	.15* (.04; .25)	.06 (−.05; .17)	.19* (.07, .30)

Abbreviations: DHD, Dutch Healthy Diet score; KA/QA, kynurenic-acid-to-quinolinic-acid ratio; HAA/HK, 3-hydroxyanthranilic-acid-to-3-hydroxykynurenine ratio; KTR, kynurenine-to-tryptophan ratio; and HKr, hydroxykynurenine ratio.

Both exposure and outcome variables are standardized.

Standard deviations of ratios are: 0.05 for KA/QA, 0.4 for HAA/HK; 10 for KTR; 0.2 for HKr; and 11 nmol/L for neopterin.

Standard deviations of dietary intake are: 15 points for DHD score; 71 g/day for vegetables; 90 g/day for fruit; 10 g/day for legumes; 63 g/day for whole grain; 7 g/day for nuts; 16 g/day for lean fish; 15 g/day for fatty fish; 264 g/day for tea; 19 g/day for alcohol; 143 g/day for dairy; 21 g/day for cheese; 14 g/day for soft fats; 12 g/day for hard fats; 51 g/day for refined grain; 27 g/day for red meat; 33 g/day for processed meat; and 115 g/day for sweetened beverages and fruit juices.

[1] Linear mixed models adjusted for age at enrollment (years), sex (male, female), renal function (μmol/L), time since end treatment (weeks), chemotherapy (yes, no), comorbidities (0, 1, ≥2), stoma (yes, no), educational level (high, medium, low), BMI (kg/m^2), total PA (hours/day), smoking status (current, former, never) and energy intake (kcal/week)

[2] The β-coefficients represent the overall longitudinal difference in kynurenine concentrations using linear mixed models, and can be interpreted as the amount of SD difference in kynurenine concentration/ratio according to 1SD difference in dietary score/intake

[3] A random slope was added to the model for vegetables with KTR; for fruit with KTR, HKr, and Neopt; for whole grain with KTR, and HKr; for nuts with KTR, HKr, HAA/HK, and Neopt; for lean fish with HKr, and Neopt; for fatty fish with HAA/HK; for tea with HKr; for alcohol with KTR, HKr, and Neopt; for dairy with HKr; for cheese with KTR; for soft fats with HKr, and Neopt; for refined grain with KTR, HKr, and HAA/HK; for red meat with KTR, and HAA/HK; for processed meat with HAA/HK, KA/QA, and Neopt; and for sweetened beverages and fruit juices with KTR, HAA/HK, and Neopt.

[4] The β-coefficients represent the change in kynurenine ratios over time within individuals using a hybrid model within linear mixed models

[5] The β-coefficients represent the difference in kynurenine ratios between individuals using a hybrid model within linear mixed models

* Indicates a statistically significant association
